# Pancreatic acinar cell carcinoma with a ductal adenocarcinoma component: a case report and analysis of the histogenesis of the tumor

**DOI:** 10.1186/s12957-020-02014-3

**Published:** 2020-09-05

**Authors:** Toshihisa Kimura, Shinsuke Tabata, Tamotsu Togawa, Hidetoshi Onchi, Atsushi Iida, Yasunori Sato, Takanori Goi

**Affiliations:** 1 Department of Surgery, National Hospital Organization Tsuruga Medical Center, 33-1 Sakuragaoka-cho, Tsuruga, 914-0195 Japan; 2 Department of Surgery, Jouhoku Hospital, 20-3 Kyomachi, Kanazawa, 920-8616 Japan; 3 Department of Surgery, Fukui General Hospital, 58-16-1, Egamimachi, Fukui, 910-8561 Japan; 4 Department of Human Pathology, Kanazawa University Graduate School of Medicine, 13-1, Takara-machi, Kanazawa, 920-8640 Japan; 5 First Department of Surgery, Faculty of Medicine, University of Fukui, 23-3, Matsuoka, Shimoaizuki, Eiheiji-cho, Yoshida-gun, Fukui, 910-1193 Japan

**Keywords:** Acinar cell carcinoma, Acinar–ductal metaplasia, Ductal adenocarcinoma, Mixed acinar carcinoma, Tumorigenesis

## Abstract

**Background:**

Pancreatic cancer composed of acinar cell carcinoma (ACC) and ductal adenocarcinoma (DAC) is rare, and the clinicopathological characteristics of ACC with DAC have yet to be elucidated. Herein, we report a case of ACC with a DAC component of the pancreas and examined the histogenesis of this tumor.

**Case presentation:**

A 69-year-old man was admitted to our hospital complaining of appetite loss, constipation, epigastric dull pain, and jaundice. Abdominal computed tomography and magnetic resonance cholangiopancreatography revealed a pancreatic head tumor with dilatation of the bile duct and the distal main pancreatic duct. Under the diagnosis of pancreatic head cancer, a pancreatoduodenectomy was performed. The histology of the resected tumor consisted of mainly ACC with a focus of DAC, which was confirmed by mucin staining and immunohistochemistry for antigens such as BCL10, trypsin, Smad4, p16, p53, and MUC1. There was histological transition between the components of ACC and DAC, and immunostaining of the transitional zone showed equivocal results for the antigens. *KRAS* was wild-type in both ACC and DAC. The patient was treated with adjuvant chemotherapy with S-1 for 1 year. No evidence of recurrence or metastasis was observed after 9 years of follow-up.

**Conclusions:**

A rare case of pancreatic ACC with a DAC component in a patient with long-term survival after surgery was reported. Immunohistochemical and molecular analysis indicated that DAC might have arisen from ACC through transdifferentiation in this case.

## Background

The pancreas is composed of exocrine and endocrine components; the exocrine components comprise ductal and acinar cells, and the endocrine components comprise endocrine cells. Most pancreatic malignancies are known to arise from a single cell type of either endocrine or exocrine origin and usually originate from ductal cells (most common), islet cells (less common), or rarely acinar cells (rare). Indeed, 95% of pancreatic exocrine tumors correspond to ductal adenocarcinoma (DAC). Acinar cell carcinoma (ACC) is rare, accounting for only 1–2% of tumors [1–3].

The prognosis of ACC remains controversial because some patients show poor prognosis, while others show a better prognosis than that of patients with DAC [4–6]. The median survival for ACC ranges from 18 to 33 months, and early detection with complete resection is reported to be beneficial to patients [4, 7, 8].

Although the frequency is low, coexistence of ACC and DAC can be encountered [1]. Due to its rarity, the clinicopathological characteristics of this type of tumor have yet to be elucidated. Herein, we report a case of ACC with a DAC component of the pancreas with long-term patient survival after surgery and examined the histogenesis of this tumor using immunohistochemical and molecular methods.

## Case presentation

**Fig. 1:**
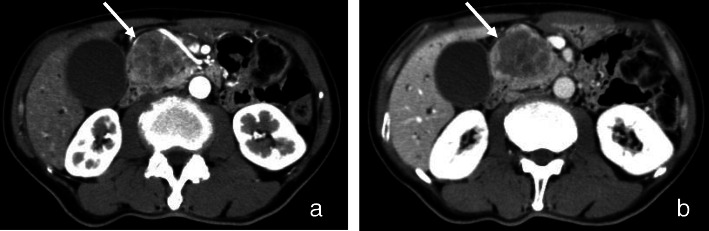
Contrast-enhanced CT revealed a slight enhancement with a poor enhanced spot in the solid tumor with an indistinct border (arrow). **a** Early phase, **b** late phase

**Fig. 2:**
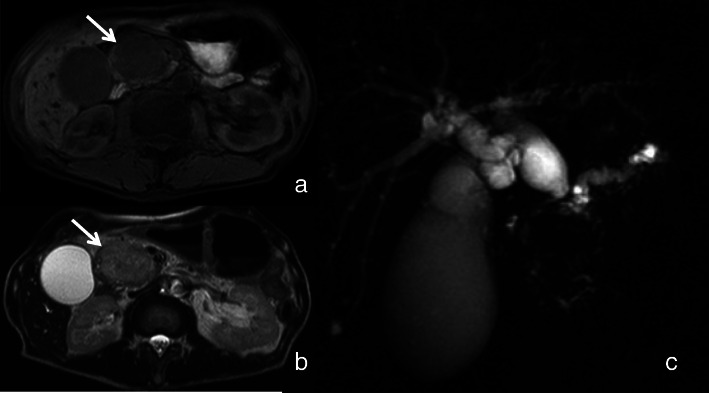
MRI showed a tumor in the pancreatic head showing low intensity on T1-weighted images (**a**) (arrow) and high-intensity images on T2-weighted images (**b**) (arrow). MRCP revealed a dilation of the main pancreatic duct from the body to the tail of the pancreas and luminal narrowing of the common bile duct (**c**)

A 69-year-old man was admitted to our hospital complaining of appetite loss, constipation, epigastric dull pain, and jaundice. His past medical history was unremarkable. He denied prior liver disease or gastrointestinal surgery. His family history was negative for liver disease and cancer. On physical examination, his height and body weight were 163.0 cm and 48.0 kg, respectively. Blood pressure was 147/95 mmHg, and body temperature was 36.8 °C. Jaundice was detected in the palpebral conjunctiva and the skin, and he had slightly tenderness on his upper abdomen. No other abnormal findings were observed. The laboratory data were as follows: serum aspartate aminotransferase, 389 IU/l (reference range, 227–416); alanine aminotransferase, 740 IU/l (227–416); γ-glutamyl transferase, 1824 IU/l (227–416); lactate dehydrogenase, 451 IU/1 (227–416); total bilirubin, 10.28 mg/dl (0.3–1.2); direct bilirubin, 6.52 mg/dl (0.05–0.3); fasting blood sugar, 275 mg/dl (70–110); hemoglobin A1c, 8.2% (4.6–6.2); amylase, 170 IU/1 (50–158); and lipase, 260 IU/1 (0–129). All other laboratory data were within normal limits. The tumor marker values were as follows: carcinoembryonic antigen (CEA), 1.2 ng/ml (reference range, < 5.9 ng/ml); carbohydrate antigen 19-9 (CA19-9), 336.6 U/ml (< 37 U/ml); DUPAN-2, 243 U/ml (< 150 U/ml); SLX, 18.8 U/ml (< 38 U/ml). Computed tomography (CT) and magnetic resonance image (MRI) revealed a pancreatic head tumor that measured 5 cm. Contrast-enhanced CT revealed a slight enhancement with a poor enhanced spot in the tumor (Fig. 1). Magnetic resonance cholangiopancreatography (MRCP) revealed a dilation of the main pancreatic duct from the body to the tail of the pancreas and luminal narrowing of the lower common bile duct (Fig. 2). On the day following hospitalization, endoscopic sphincterotomy and endoscopic retrograde biliary drainage with a 7-Fr, 7 cm pigtail plastic stent were performed. At 4 weeks following the procedures, all tumor markers (CEA, CA19-9, DUPAN-2, and SLX) were within the normal range. CT revealed that the size of the pancreas head increased to 6.5 × 5.2 × 4.5 cm, but there was no sign of invasion in the main blood vessels, remote transition, peritoneal metastasis, or lymph node metastasis.

The patient was diagnosed with pancreatic head cancer, and a pancreatoduodenectomy reconstructed by Child’s procedure with resection of regional lymph nodes was performed. Neither liver metastasis nor peritoneal metastasis was observed, and lymph node metastasis was not readily identified intraoperatively.

### Pathological findings

**Fig. 3:**
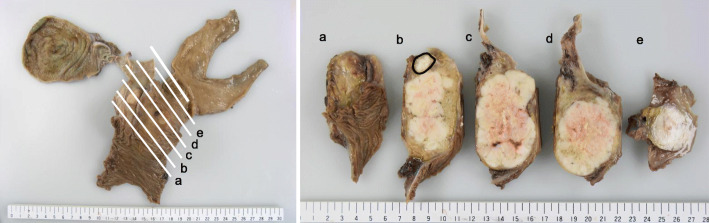
Macroscopic view of the resected specimen. A solid tumor in the pancreatic head, in which the vast majority of the tumor was histologically composed of acinar cell carcinoma. The area corresponding to the focus of ductal adenocarcinoma and/or transitional zone is indicated by a black circle

The resected specimen clearly showed a large tumor located in the pancreatic head (Fig. 3). The cut surface of the tumor was gray-white in color, measuring 7.0 cm in diameter at the widest point. It showed expansive growth and invaded the distal bile duct in the pancreatic head.

**Fig. 4:**
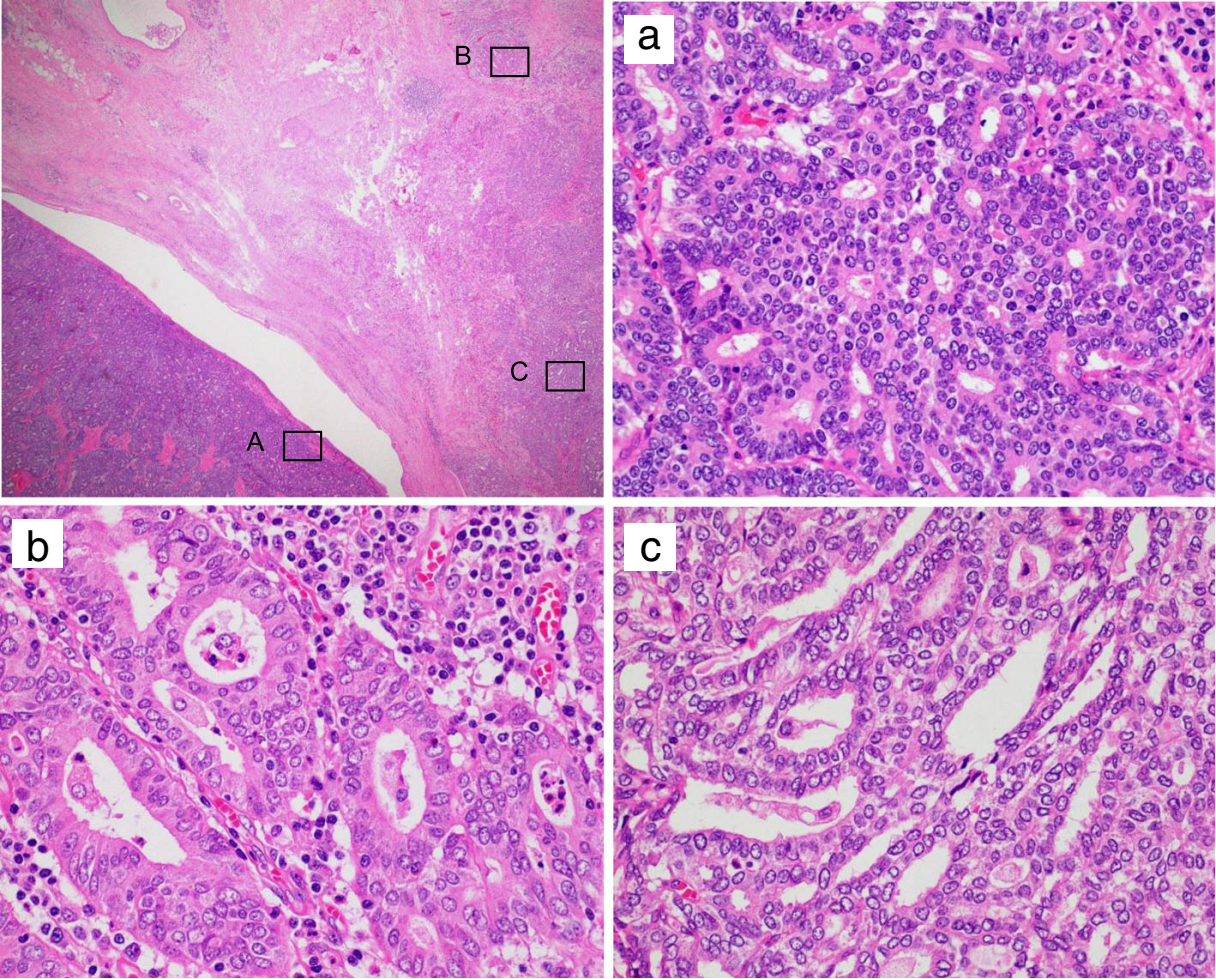
Histology of the pancreatic tumor. The tumor was composed mainly of acinar cell carcinoma (**a**). At the periphery of the tumor, there was a focus of ductal adenocarcinoma (**b**). Between the two components, a transitional zone was observed (**c**). Hematoxylin and eosin staining

**Fig. 5:**
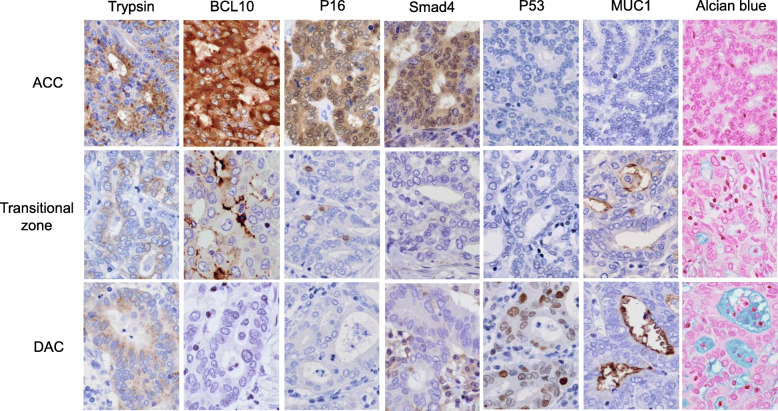
Results of immunostaining and mucin staining. Acinar cell carcinoma (ACC) showed positive staining of acinar cell markers, trypsin, and BCL10. The component of ductal adenocarcinoma (DAC) showed positivity for ductal markers, MUC1, and Alcian blue. In the DAC component, the expression of p16 and Smad4 was lost, and p53 was expressed. The transitional zone showed equivocal results

Histologically, the tumor consisted of an admixture of two different components: ACC and DAC (Fig. 4). The ACC component was predominant and accounted for more than 90% of the entire tumor. In the ACC component, the tumor showed acinar, cribriform, or solid patterns of growth with no or little fibrous stroma, and necrotic tendency was focally observed. In the DAC component, the tumor showed a glandular growth pattern with desmoplastic fibrous stroma associated with inflammation, and mucin production was detected by staining with Alcian blue pH 2.5 (Fig. 5). The focus of DAC was located at the periphery of the tumor, and histological transition was observed between the components of ACC and DAC. The foci of pancreatic intraepithelial neoplasia (PanIN) were not observed in the pancreatic ducts. Nodal metastasis was found in one of 35 dissected lymph nodes.

Immunohistochemical analysis was performed using primary antibodies listed in Table 1. The results of immunostaining as well as mucin staining were graded as follows: − (negative), + (focally or weakly positive), and ++ (diffusely positive). On immunohistochemical staining, the tumor cells of ACC showed diffuse positivity for acinar cell markers, trypsin, and BCL10 and negativity for the ductal marker MUC1 (Table 2, Fig. 5). In contrast, the tumor cells of DAC were positive for MUC1 and p53, and focal weak positivity for trypsin was also observed. The expression of BCL10 was not observed, and the expression of p16 and Smad4 was lost in the DAC component. These results confirmed the histological diagnosis of ACC and DAC. The transitional zone of ACC and DAC displayed equivocal immunohistochemical results for these markers. The metastatic tumor in the lymph node showed glandular structure with positive immunoreactivity for MUC1, indicating DAC origin. The analysis of *KRAS* mutations was conducted in the laboratory of a private contracting company (BML, Inc.; Tokyo, Japan), and the results showed that *KRAS* mutations at codons 12 and 13 were not observed in either component of ACC or DAC.

**Table 1:** Primary antibodies used for immunostaining

Antibody	Clone	Source	Dilution
BCL10	331.3	Santa Cruz Biotechnology (Santa Cruz, CA)	1:50*
MUC1	DF3	Toray Fuji Bionics (Tokyo, Japan)	1:50**
P16	E6H4	Roche (Basel, Switzerland)	Prediluted**
P53	DO-7	Dako; Agilent Technologies (Tokyo, Japan)	1:100**
Smad4	B-8	Santa Cruz Biotechnology	1:50**
Trypsin	Monoclonal	Millipore; Merck (Darmstadt, Germany)	1:2000**

In immunohistochemical staining, antigen retrieval was performed by heating in tris-ethylenediaminetetraacetic acid buffer (pH 8.5) (*) or by microwaving in 10 mmol/L citrate buffer (pH 6.0) (**)

**Table 2:** Summary of the results of immunostaining and mucin staining

	Trypsin	BCL10	P16	Smad4	P53	MUC1	Alcian blue
ACC	++	++	++	++	−	−	−
Transitional zone	+	+	+	−	−	+	+
DAC	+	−	−	−	+	++	++

*++* diffusely positive, *+* focally or weakly positive, *−* negative. *ACC* Acinar cell carcinoma, *DAC* Ductal adenocarcinoma

The World Health Organization (WHO) categorizes mixed acinar–ductal carcinoma as a subtype of ACC, and it is defined as having > 30% of each line differentiation [9]. In this case, since the DAC component accounted for less than 10% of the tumor, the tumor was pathologically diagnosed as ACC with a DAC component of the pancreas. The TNM classification according to UICC was conclusive in T2, N1, M0, and stage II B.

### Postoperative course

The postoperative course was uneventful. The patient was treated with adjuvant chemotherapy with S-1 for 1 year. No evidence of recurrence or metastasis was observed after 9 years of follow-up.

## Discussion

Mixed acinar carcinomas of the pancreas listed in the WHO classification include mixed acinar-ductal carcinoma, mixed acinar-neuroendocrine carcinoma, and mixed acinar-neuroendocrine-ductal carcinoma [9]. Because of the limited number of cases, the clinicopathological and genomic features of these mixed acinar carcinomas are poorly understood, and there is still considerable uncertainty regarding the recurrence form and prognosis.

The radiological features of these tumors also remain unclear. Preoperative diagnostic imaging of mixed acinar–ductal carcinoma may reflect imaging findings for ACC and DAC. On contrast CT, ACC usually displays a relatively larger size, exophytic growth, a well-defined margin with enhanced capsule, a lack of or relatively mild pancreatic duct dilatation or vascular encasement, internal necrosis, and cystic changes, whereas DAC appears as a solid tumor with poor vascularity and pancreatic duct dilatation or vascular encasement [10]. In the present case, a slight enhancement with a poor enhanced spot in the tumor with an indistinct border was observed on contrast-enhanced CT.

Preoperative diagnosis of mixed acinar–ductal carcinoma is difficult because the imaging findings depend on the ratio of the ACC and DAC components, which differs from case to case. Concerning preoperative definitive diagnosis, endoscopic ultrasonography-guided fine needle aspiration is an excellent tool to obtain a histological diagnosis, but a precise diagnosis can be difficult when the tumor has compositional heterogeneity [11].

Medical treatments for mixed acinar–ductal carcinoma have not been established. For ACC, aggressive surgical resection with the goal of achieving R0 margins of resection is associated with long-term survival [12, 13]. Even after curative resection, there is still a high rate of recurrence [4, 9]. It is inferred from the good postoperative prognosis of our patient that surgical resection may be the first choice of medical treatment when there is no non-excising factor. R0 excision is also thought to improve the prognosis of cases.

The chemotherapeutic effects of gemcitabine on DAC are established, but its effects on ACC have not been well documented. In this context, the chemotherapy effects of gemcitabine for mixed acinar–ductal carcinoma may depend on the area of the DAC component within the tumor, as it may be more effective for tumors with a predominant DAC component.

DAC develops through the multistep carcinogenic process, and PanIN is known as a precancerous lesion [14, 15]. Cells in the pancreas that have undergone acinar–ductal metaplasia can transform to premalignant cells that can eventually become cancerous [16]. In a mouse model of genetically induced pancreatic cancer, activating *KRAS* mutations induces the differentiation of acinar cells to duct-like cells and progression to PanIN [17]. During the progression from premalignant lesion to invasive carcinoma, *KRAS* mutations are the initial events where > 90% of PanIN lesions harbor these mutations, followed by the accumulation of tumor suppressive genes such as p16, Smad4, and TP53 [9, 18]. It has been reported that ACC does not typically harbor mutations in such genes [19]. On immunohistochemical staining, for example, p53 expression is altered in 75–80% in DAC with either nuclear overexpression or no expression (null type), whereas the nuclear overexpression of p53 is reported to be observed only in 21% of ACC cases [9, 20].

For tumorigenesis, we considered four possible mechanisms in this case: (1) ACC arose from DCC through transdifferentiation; (2) DCC arose from ACC through transdifferentiation; (3) both ACC and DCC developed from the same cancer stem cell; and (4) ACC and DCC developed independently (collision tumor). In this case, immunohistochemical analysis showed the loss of expression of p16 and Smad4 and the overexpression of p53 in the DAC component. In contrast, alterations in the expression of these proteins were not detected in the ACC component. These findings indicated that ACC might not arise from DAC because in such circumstances, the altered expression of p16, Smad4, and p53 must be observed in the ACC component.

In addition, the collision tumor was unlikely because histological transition was observed between the components of ACC and DAC. Similarly, if ACC and DAC developed from the same cancer stem cell through the biphasic differentiation process, the presence of the transitional area appeared to be agonistic. Taken together, we suggest that DAC arose from the ACC through transdifferentiation, i.e., ACC developed first, and it acquired features characteristic of DAC, such as alterations in the expression of p16, Smad4, and p53. The equivocal immunohistochemical results of the transitional zone between ACC and DAC and the absence of PanIN and *KRAS* mutations were consistent with this suggestion.

Pancreatic ductal cells can transform to squamous epithelium via metaplasia in conditions such as chronic pancreatitis. Recently, it has been reported that the squamous carcinoma component of pancreatic adenosquamous carcinoma originates from preexisting DAC via transdifferentiation [21]. Similarly, it is not surprising that ACC can transdifferentiate into DAC because acinar cells originally have the ability to transform into duct-like cells via acinar–ductal metaplasia.

## Conclusions

A rare case of ACC with a DAC component of the pancreas in a patient with long-term survival after surgery was reported. The immunohistochemical analysis as well as *KRAS* mutation analysis indicated that DAC might have arisen from ACC through transdifferentiation, although further case studies are required to clarify the histogenesis of this rare type of tumor.

## Data Availability

All data generated or analyzed during this study are included in this published article.

## Abbreviations

ACC: Acinar cell carcinoma
CT: Computed tomography
CEA: Carcinoembryonic antigen
CA19-9: Carbohydrate antigen 19-9
DAC: Ductal adenocarcinoma
MRCP: Magnetic resonance cholangiopancreatography
MRI: Magnetic resonance image
PanIN: Pancreatic intraepithelial neoplasia
UICC: Union for International Cancer Control
WHO: World Health Organization
